# Supplementary figures and images for: Phenotypic, Chemotaxonomic, and Genome-Based Classification of Phyllobacterium Strains: Two Proposed Novel Species, Phyllobacterium chamaecytisi sp. nov. and Phyllobacterium lublinensis sp. nov
Source: Biology (Basel). 2025 Aug 8;14(8):1024. doi: 10.3390/biology14081024 (PMC12383984; doi:10.3390/biology14081024)

**A.** *Phyllobacterium lublinensis* 2063

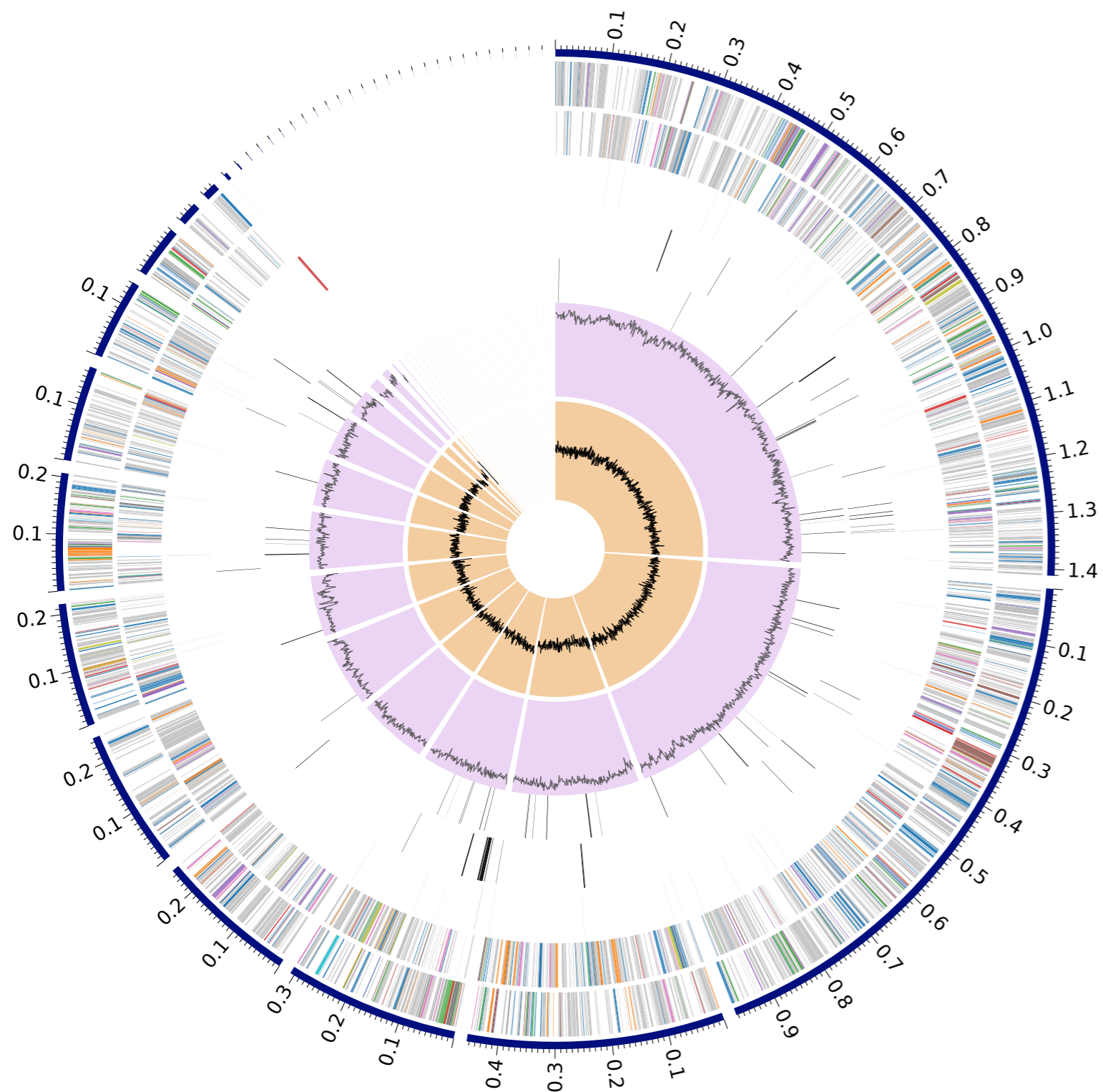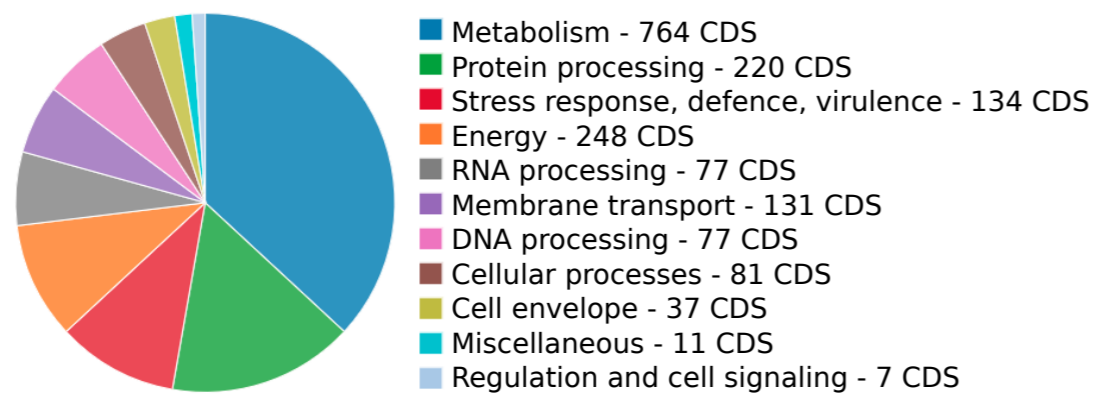

**B.** *Phyllobacterium chamaecytisi* KW56

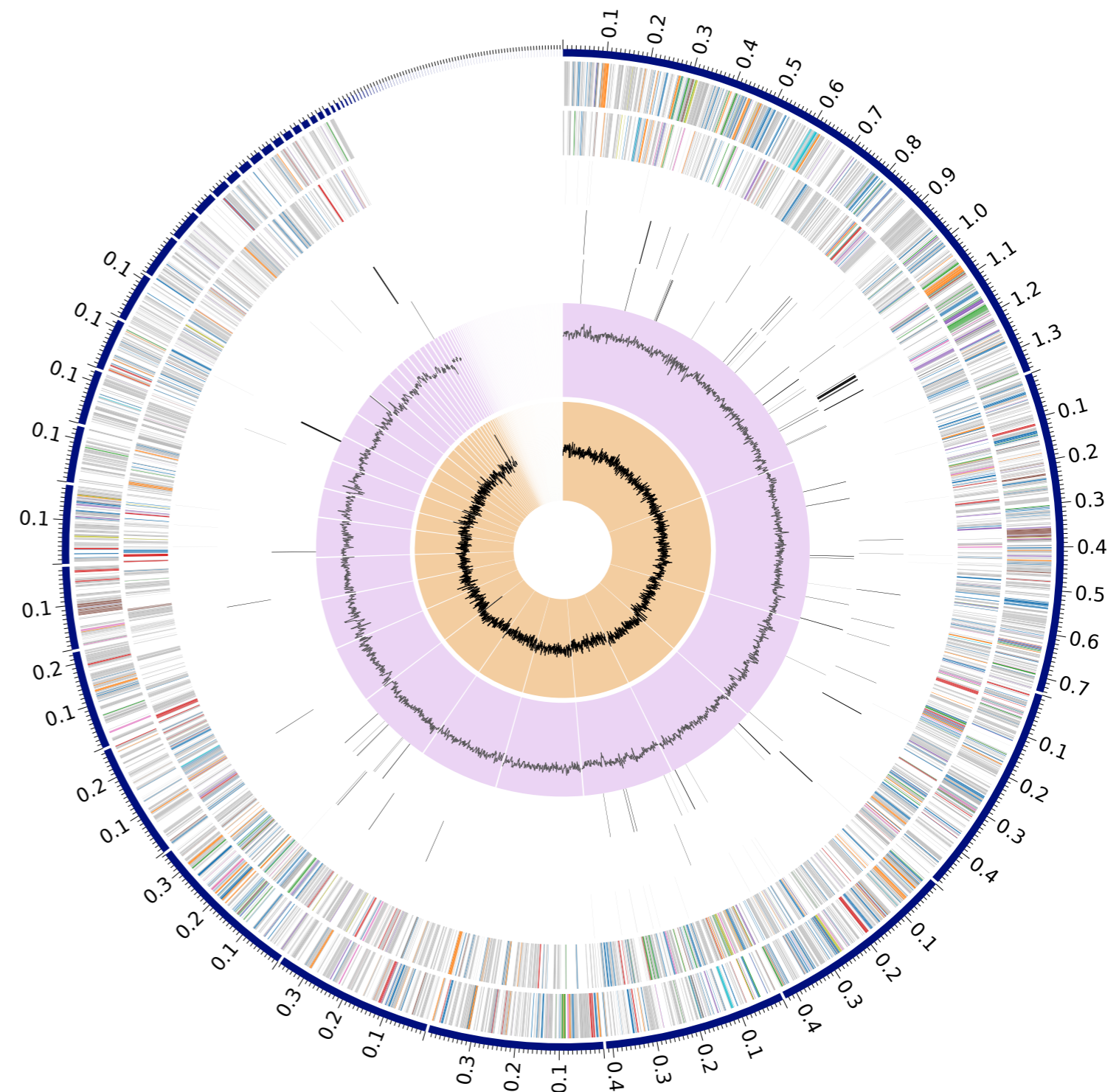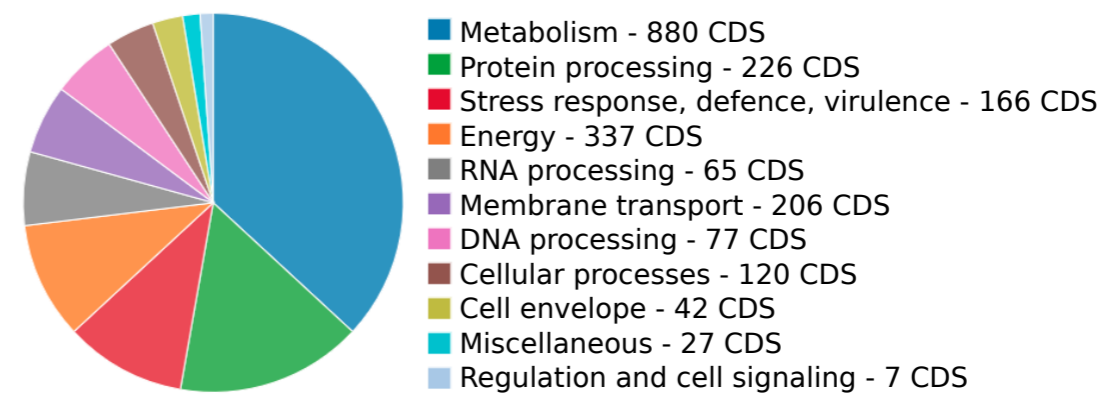

Supplement: Supplementary file 1 [file biology-14-01024-s001.zip › biology-3698617-supplementary.pdf]
